# P068. A suggestive case of hemiplegic migraine: a diagnostic challenge

**DOI:** 10.1186/1129-2377-16-S1-A82

**Published:** 2015-09-28

**Authors:** Emanuela Garrone, Corinna Garrone, Antonella Versace, Carlotta Canavese, Margherita Conrieri, Giuseppina Migliore, Emanuele Castagno, Rosaura Pagliero

**Affiliations:** Centro Cefalee dell'età evolutiva, S.C. Pediatria d'Urgenza, Ospedale Infantile Regina Margherita, AOU Città della Salute e della Scienza, Turin, Italy; S.C.D.U. Neuropsichiatria Infantile, Ospedale Infantile Regina Margherita, AOU Città della Salute e della Scienza, Turin, Italy

## Introduction

We describe a case of possible hemiplegic migraine (HM), whose diagnosis was challenging and required exclusion of other pathologies including ischemic and inflammatory diseases.

## Case report

A 12-year-old boy was referred to our Emergency Department because of fever and temporal headache with transitory visual aura, nausea, vomiting, unilateral motor clumsiness since the day before. In his medical history, some episodes of mild headache were reported; he had family history of recurrent headache (mother and aunt). At arrival he presented a discrete general condition, paleness and fever. He was restless and confused, only partially collaborative, able to answer just simple questions and presenting left hypostenia, mild left hypoestesia and left hyporeflexia. Blood tests proved negative. Cerebral computed tomography and cerebral magnetic resonance with vascular study were all negative. The cerebrospinal fluid test showed rare leukocytes. The electroencephalographic (EEG) showed diffuse signs of suffering with prevalence at the right temporal areas, thus antiviral and cortisonic therapy were started, along with antibiotics. Further investigations included the thrombophilia panel and trans-cranial echo-color-doppler study with echocontrast, all resulted negative. During admission, the child remained afebrile with rapid resolution of headache intensity and neurological involvement; an EEG repeated two days later was normal. The clinical course and laboratory/imaging results excluded both the infectious and the ischemic hypotheses. The results of the genetic analysis of CACNA1A, SCN1A, ATP1A2 genes are still ongoing.

## Conclusions

Our patient presented a single episode of fully reversible motor weakness with fully reversible visual and sensory symptoms, associated with headache, whose sequence of events and features is suggestive of HM, although one single episode is not sufficient for diagnosis, according to the ICHD3-beta diagnostic criteria[1, 2]. In such cases, the meticulous description of aura and urgent and exhaustive investigations to search for all possible alternative causes (including stroke, tumors, infectious or inflammatory diseases, all excluded in our patient) are mandatory. Genetic tests are available to confirm the diagnosis, and in our case the result is still not available[1, 3].

Written informed consent to publication was obtained from the patient(s).
